# Rapid Antiretroviral Therapy Initiation for Women in an HIV-1 Prevention Clinical Trial Experiencing Primary HIV-1 Infection during Pregnancy or Breastfeeding

**DOI:** 10.1371/journal.pone.0140773

**Published:** 2015-10-15

**Authors:** Susan Morrison, Grace John-Stewart, John J. Egessa, Sezi Mubezi, Sylvia Kusemererwa, Dennis K. Bii, Nulu Bulya, Francis Mugume, James D. Campbell, Jonathan Wangisi, Elizabeth A. Bukusi, Connie Celum, Jared M. Baeten, Partners PrEP Study Team

**Affiliations:** 1 Department of Global Health, University of Washington, Seattle, WA, United States of America; 2 Department of Medicine, University of Washington, Seattle, WA United States of America; 3 Department of Epidemiology, University of Washington, Seattle, United States of America; 4 The AIDS Support Organization, Kampala, Uganda; 5 Infectious Disease Institute, Makerere University, Kampala, Uganda; 6 Center for Microbiology Research, Kenya Medical Research Institute, Nairobi, Kenya; 7 Kabwohe Clinical Research Center, Kabwohe, Uganda; 8 Centers for Disease Control and Prevention, Entebbe, Uganda; University of Missouri-Kansas City, UNITED STATES

## Abstract

During an HIV-1 prevention clinical trial in East Africa, we observed 16 cases of primary HIV-1 infection in women coincident with pregnancy or breastfeeding. Nine of eleven pregnant women initiated rapid combination antiretroviral therapy (ART), despite having CD4 counts exceeding national criteria for ART initiation; breastfeeding women initiated ART or replacement feeding. Rapid ART initiation during primary HIV-1 infection during pregnancy and breastfeeding is feasible in this setting.

## Introduction

Primary HIV-1 infection carries a high risk of maternal-to-child HIV-1 transmission (MTCT) when occurring during pregnancy or breastfeeding [1, 2]. HIV-1 acquisition during pregnancy is often not identified because of limited repeat HIV-1 testing, although high HIV-1 incidence during this period has been observed [2, 3, 4]. Initiation of antiretroviral therapy (ART) reduces the risk of MTCT; however, few countries have policies which specifically address primary HIV-1 infection during pregnancy or breastfeeding or prioritize treatment in this situation. Women with primary HIV-1 infection are likely to have higher CD4 counts and thus not qualify for combination ART under many national guidelines for routine ART initiation, or may not be prioritized for urgent initiation where policies for universal treatment during pregnancy (Option B/B+) have been adopted.

Randomized clinical trials of novel HIV-1 prevention strategies frequently enroll reproductive-age women at high HIV-1 risk, who may also become pregnant during study follow-up. Frequent, scheduled HIV-1 testing presents an opportunity to identify HIV-1 acquisition, potentially permitting initiation of combination ART to reduce the risk of MTCT. However, HIV-1 prevention trial sites do not typically provide direct HIV-1 care, instead referring HIV-1 seroconverters to local institutions.

## Methods

Within a randomized clinical trial of PrEP for HIV-1 prevention, we identified 16 cases of HIV-1 acquisition among pregnant or postpartum women and, working with local institutions, were able to rapidly initiate ART, including for women with CD4 counts above the thresholds for routine ART initiation.

Between July 2008 and December 2012, we conducted the Partners PrEP Study, a randomized, placebo-controlled trial of daily oral tenofovir and emtricitabine/tenofovir PrEP among 4747 HIV-1 uninfected members of HIV-1 serodiscordant couples in Kenya and Uganda. Eligibility criteria, study procedures, and trial results have been reported previously [5]. Participants were tested monthly for HIV-1 using paired rapid tests, confirmed by HIV-1 EIA if positive, and female participants had monthly urine pregnancy testing. The study medication was discontinued when pregnancy was identified, but pregnant women continued with monthly visits, including HIV-1 testing. A primary criterion in site selection was established linkages with HIV-1 care centers. Participants who acquired HIV-1 were referred to local partnering HIV-1 care centers, including government health facilities and non-governmental organizations (NGOs), where ART was provided according to national guidelines during and after study participation. Women who acquired HIV-1 while pregnant or breastfeeding were referred expediently with direct communication with the partnering institutions.

## Ethics Statement

The Partners PrEP Study was approved by the University of Washington Human Subjects Review Committee and ethics review committees for each study site, specifically the Moi University Institutional Research and Ethics Committee and Indiana University Human Subjects Office (Eldoret, Kenya site), Office of Research Administration, Kenya Medical Research Institute Ethics Review Committee and the University of California—San Francisco Committee for Human Research (Kisumu, Kenya site), Kenyatta National Hospital Ethics and Research Committee (Nairobi and Thika, Kenya sites), the National HIV/AIDS Research Committee of the Uganda National Council for Science and Technology (Jinja, Kabwohe, and Kampala, Uganda sites), and the Uganda Virus Research Institute Science and Ethics Committee and the Centers for Disease Control and Prevention Human Research Protection Office (Mbale and Tororo, Uganda sites). All study participants provided written informed consent to participate in English or their local language, and the consent documents were approved by the overseeing ethics committees.

## Results

During the study, 70 women seroconverted to HIV-1, 16 of whom (23%) experienced primary HIV-1 infection concurrently with pregnancy or breastfeeding. Of these, 11 (16%) were pregnant at or within three months after the time of HIV-1 seroconversion, and five (7%) were breastfeeding at the time of seroconversion (Table 1). Cases occurred at six of nine study sites.

**Table 1:** Participants with primary HIV-1 infection identified during pregnancy or breastfeeding.

Country date	Age	Timing of HIV-1 seroconversion	CD4 count (cells/μL)	Immediate intervention to reduce HIV-1 transmission risk, time to intervention *	Infant outcome
**HIV-1 seroconversion during pregnancy**					
KenyaAug 2009	23	9 2/7 weeks prior to pregnancy identification	978	Combination ART (zidovudine, lamivudine, lopinavir/ritonavir),27 days	Term live birth. HIV-1 DNA PCR negative at 6 weeks of age
KenyaOct 2010	23	3 5/7 weeksof pregnancy	1053	Referred for combination ART but lost to follow-up until 19 2/7 weeks of pregnancy, then zidovidine monotherapy	Preterm birth, neonatal death
Uganda Nov 2010	27	34 6/7 weeks of pregnancy	604	Combination ART (zidovudine, lamivudine, nevirapine),11 days	Term live birth. HIV-1 DNA PCR negative at 8 weeks of age
Uganda Jan 2011	28	31 4/7 weeks of pregnancy	662	Combination ART (zidovudine, lamivudine, nevirapine),19 days	Term live birth. HIV-1 DNA PCR negative at 4 months of age
UgandaJan 2011	30	14 4/7 weeksof pregnancy	473	Combination ART (zidovudine, lamivudine, lopinavir/ritonavir),43 days	Term live birth. HIV-1 DNA PCR negative at 13 months of age
Uganda Jan 2011	25	15 6/7 weeks of pregnancy	824	Declined ART	Induced abortion
UgandaMar 2011	25	13 5/7 weeks of pregnancy	306	Combination ART (zidovudine, lamivudine, tenofovir),11 days	Term live birth. HIV-1 DNA PCR negative at 6 weeks of age
UgandaMar 2011	34	25 1/7 weeks of pregnancy	459	Combination ART (zidovudine, lamivudine, efavirenz),26 days	Term live birth. HIV-1 DNA PCR negative at 7 months of age
Uganda Mar 2011	27	12 3/7 weeks prior to pregnancy identification	900	Combination ART (zidovudine, lamivudine, efavirenz),13 days	Term live birth. HIV-1 DNA PCRnegative at 6 months of age
UgandaJul 2011	24	35 2/7 weeks of pregnancy	529	Combination ART (zidovudine, lamivudine, nevirapine),2 days	Term live birth. HIV-1 DNA PCR negative within 1 week of age
Uganda Sep 2012	37	27 1/7 weeks of pregnancy	797	Combination ART(lamivudine, tenofovir, efavirenz),7 days	Term live birth. HIV-1 DNA PCR negative at 2 months of age
**HIV-1 seroconversion during breastfeeding**					
Uganda Jun 2010	26	Breastfeeding 14 weeks	963	Replacement feeding,5 days	HIV-1 DNA PCR negative within 2 weeks of maternal seroconversion
Uganda Apr 2012	28	Breastfeeding 12 weeks	524	Combination ART (zidovudine, lamivudine, nevirapine),10 days	HIV-1 DNA PCR negative within 2 weeks of maternal seroconversion.
UgandaJul 2012	27	Breastfeeding 12 months	422	N/A.Infant HIV-1 infected	HIV-1 DNA PCR positive within 2 weeks of maternal seroconversion
Uganda Sep 2012	32	Breastfeeding 17 months	738	Breastfeedingcessation, 0 days	HIV-1 DNA PCR negative 7 months after maternal seroconversion
UgandaOct 2012	29	Breastfeeding 12 months	813	Breastfeeding cessation,0 days	HIV-1 DNA PCR negative within 2 weeks of maternal seroconversion

* Time to intervention is measured from the date of the first positive rapid HIV-1 test until antiretroviral therapy is initiated. For those who seroconverted to HIV-1 prior to pregnancy, time to intervention is measured from the date of the first positive pregnancy test until antiretroviral therapy is initiated.

Among the 11 women with concurrent pregnancy and seroconversion, nine initiated combination ART (82%). Eight of the nine women had CD4 counts >350 cells/μL. One participant declined therapy and terminated her pregnancy, and a second was lost to follow-up prior to ART initiation. For the nine pregnant participants starting combination ART, the median time to initiation was 13 days (range 2–43 days). All nine infants underwent HIV-1 DNA PCR testing after birth and results were negative.

Four of the five participants who were breastfeeding at the time of maternal seroconversion received immediate intervention to prevent MTCT, individualized according to infant age, existing national guidelines, available services, and wishes of the parents. One participant rapidly initiated combination ART while continuing to breastfeed her three month-old infant. One chose replacement feeding and received intensive support. Two weaned older infants (12 and 17 months) and received nutrition counseling. These four infants had negative initial HIV-1 DNA PCR results. The final participant’s infant had HIV-1 DNA PCR testing at the time of maternal seroconversion and was found to be already HIV-1 infected; breastfeeding was continued in addition to referral of the infant for care.

## Discussion

In summary, within this randomized clinical trial of an HIV-1 prevention intervention, we prospectively identified 16 pregnant or breastfeeding women with primary HIV-1 infection. Study personnel worked closely with participants and local partners to ensure successful linkage to care and ART. Ten women initiated combination ART, six within two weeks of the positive HIV-1 test, and nine of the ten within 30 days.

At the time of this study, Kenya and Uganda national guidelines recommended combination ART for those whose CD4 counts was <200–350 cells/mm^3^ (with the higher CD4 threshold phased in during the study period), with short course therapy for the prevention of MTCT recommended for women who did not meet criteria for full ART initiation, and without specific provision for ART during primary HIV-1 for prevention of MTCT. Thus, ART initiation in all ten cases in our study was for an indication beyond the scope of national guidelines. In one case, ART was not initiated until 43 days after HIV-1 seroconversion was detected due to initial reluctance of the partnering institution to operate beyond national guidelines.

While the research setting we describe is unique, our findings are generalizable to maternal and child health programs in which primary HIV-1 infection may be identified among initially-HIV-1-seronegative women offered retesting later in pregnancy during antenatal care visits or postpartum at maternal, infant, and child health clinics. The response we observed from local providers suggests that ART initiation in primary infection during pregnancy among mothers at high risk of transmitting HIV-1 to their infants was well received. Retesting in pregnancy is recommended by WHO in settings of generalized epidemics [6], although this may be infrequent in practice and thus, women with primary infection may have delayed diagnosis and receive routine, rather than urgent treatment. WHO-recommended Option B/B+, which includes combination ART for all HIV-1 infected pregnant women regardless of CD4 count, is being implemented in several African countries, and would include ART initiation for women with primary HIV-1 [7].

An HIV-1 prevention clinical trial, in which HIV-1 testing is frequent, allows detection of primary HIV-1 infection and presents an opportunity for secondary prevention through rapid initiation of combination ART to prevent infant HIV-1 infection. Our results demonstrate that collaboration between a clinical trial site and partnering HIV-1 care providers can result in pregnant and postpartum women promptly initiating ART during primary HIV-1 infection. Increased expediency is recommended and could be achievable, particularly in settings where on-site provision of antiretroviral therapy is available. In this cohort, nearly 25% of women who seroconverted to HIV-1 were pregnant or breastfeeding during primary infection, underscoring the importance of HIV-1 prevention strategies which are safe and effective for reproductive age women. HIV-1 prevention trials should incorporate mechanisms to ensure women with concurrently-identified pregnancy or breastfeeding and primary HIV-1 infection are offered ART rapidly to reduce the high risk of vertical transmission.
